# Novel Copper Photoredox Catalysts for Polymerization: An In Situ Synthesis of Metal Nanoparticles

**DOI:** 10.3390/polym12102293

**Published:** 2020-10-07

**Authors:** Haja Tar, Tahani I. Kashar, Noura Kouki, Reema Aldawas, Bernadette Graff, Jacques Lalevée

**Affiliations:** 1 Department of Chemistry, College of Science, Qassim University, King Abdulaziz Rd, Buraydah, Qassim 1162 SA, Saudi Arabia; n.kouki@qu.edu.sa (N.K.); Rdoas@qu.edu.sa (R.A.); 2 Department of Chemistry, Faculty of Science, Menoufia University, Shebin El-Kom 32511, Egypt; tahanikashar@yahoo.com; 3 Institut de Science des Matériaux de Mulhouse IS2M – UMR CNRS 7361 – UHA, 15, rue Jean Starcky, 68057 Mulhouse CEDEX, France; bernadette.graff@uha.fr (B.G.); jacques.lalevee@uha.fr (J.L.)

**Keywords:** copper complex, 2,4 dinitrophenylhydrazone ligand, free radical polymerization, photoredox catalysts, free radical polymerization, gold nanoparticles

## Abstract

The copper II complex (HLCuCl) carrying 2,4 dinitrophenylhydrazone (L) is synthesized and evaluated as a new photoredox catalyst/photoinitiator in combination with triethylamine (TEA) and iodonium salt (Iod) for the radical polymerization of ethylene glycol diacrylate during exposure to visible light using a photoreactor at 419 nm. The copper complex reactivity with TEA/Iod salt/gold chloride showed a good production and stability of gold nanoparticles. Finally, the high performance of Cu (II) complex for radical photopolymerization incorporating gold nanoparticles is provided. The photochemical mechanisms for the production of initiating radicals are studied using cyclic voltammetry. Polymer nanocomposites containing gold nanoparticles (Au NPs) in situ photogenerated during the irradiation process were prepared. The formation of Au NPs inside the polymer matrix was through UV–Vis and EDS/SEM analyses.

## 1. Introduction

Metal nanoparticle-polymer composites have seen intense research interest in recent years [1,2,3,4,5]. A polymer matrix in which nanoparticles are embedded to enhance some particular properties of the parent matrix is called a polymer nanocomposite. In this method, metal ions are reduced inside a polymer matrix. This technique leads to the homogeneous dispersion of metallic nanoparticles in the polymer matrix [6]. In the past, there has been a lot of work done by different scientists to achieve this specific type of polymer nanocomposite [7,8,9,10,11,12,13,14]. The photochemical synthesis method of gold NPs has attracted a lot of attention because it offers several advantages—for example, convenience, speed, and spatial selectivity. Therefore, the photopolymerization method is widely used for the preparation of nanocomposite materials because it allows the rapid formation of polymer networks with new properties [15]. Nano-coatings are generally prepared by UV irradiation formulations containing dispersed nanoparticles [16]. An in situ photo-polymerization process was used to prepare Ag and Au nanoparticles, as reported in the literature [17,18,19,20,21,22,23,24].

Photopolymerizable formulations generally consist of photoinitiators, oligomers, and additives. Among these constituents, the photoinitiator plays an important role in the final properties of the cured coating, as well as in those of reactive oligomers and diluents. Metal complexes (zinc, ruthenium, iridium, and copper) have been used as photoinitiators for radical and cationic polymerization [25,26,27,28,29,30].

These photoinitiators possess an excellent photochemical property (e.g., the intense absorption of visible light, long-lived excited states, and suitable redox potentials) and can function through an oxidation or reduction cycle to produce reactive species—e.g., radicals, anions, or cations. Recently, copper complexes have been gaining more and more attention in the field of photopolymerization due to their comparative cost advantage. Copper complexes have received increasing attention due to their relative cost, and over the last few years extensive efforts have been devoted to developing copper-based photoinitiating systems (PISs) to initiate photointiated polymerization [29,30,31].

On the one hand, we want to explore the role of new hydrazone ligand in copper II complex used as a photoredox catalyst for polymerization reactions. On the other hand, this later will be tested as a reducer for gold III to gold nanoparticles. Actually, these have attracted scientific interest due to their broad pharmacological profile, including their antitumor, antimicrobial, and antitubercular potential [32,33,34,35,36]. The new copper II complex was incorporated into PIS (containing iodonium salt, triethyl amine, and gold (III) chloride( to generate species (i.e., radicals and cations) and gold nanoparticles inside the polymer matrix. The photoinitiation ability of the copper II complex-based PISs for the radical polymerization and synthesis of networks of interpenetrated gold nanoparticles polymers under light at 419 nm (photoreactor) with an intensity of irradiation of 250 microwatts/cm^2^ will be studied. The reactivity of the system and the photochemical efficiency will be discussed.

## 2. Materials and Methods

### 2.1. Materials

**Scheme 1:**
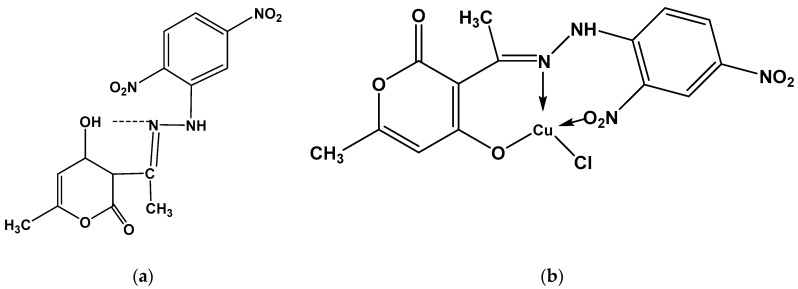
Structures of the ligand (**a**) and copper complex (**b**) used in this study.

The synthesis of this new hydrazone ligand and its copper complex is presented in detail in the supporting information. Remarkably, ligand and its copper complex are completely new (see Scheme 1).

**Scheme 2:**
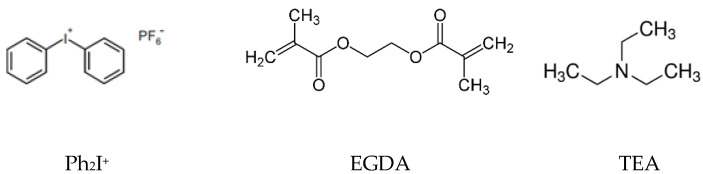
Chemical structures of the monomers and additives used in this study.

Triethylamine (TEA), gold (III) chloride HAuCl_4_, dimethylformamide (DMF), ethylene glycol diacrylate (EGDA), and diphenyliodonium hexafluorophosphate (Ph_2_I^+^) were obtained from Sigma Aldrich. The following Scheme 2 shows the structure of the chemical compounds used in the photopolymerization processes.

### 2.2. Irradiation Source

The solution was put into a Pyrex tube ((i.d.) 9 mm) and irradiated in a photochemical reactor consisting of a 35 W LED lamp at 419 nm with an irradiation intensity of 250 microwatts/cm^2^ without a water cooling system under an air atmosphere at room temperature.

### 2.3. Free Radical Photopolymerization

The three-component photoinitiating systems are mainly based on HLCuCl/TEA/Iodonium salt (0.05/1%/1% *w*/*w*), to the gold (III) chloride added 4wt% in a few drops of DMF, and the PIS system was dissolved in EGDA at 93.95 wt%. The weight percent of the photoinitiating system was calculated from the monomer content. The evolution of the SPR nanoparticles was continuously followed by a Shimadzo UV-1800 spectrophotometer (Shimadzo, Duisburg, Germany). The polymer/composite obtained is characterized by SEM.

### 2.4. Redox Potentials

The Cu complex oxidation potentials (E_ox_ vs. SCE) were measured in acetonitrile by cyclic voltammetry with tetrabutyl-ammonium hexafluorophosphate 0.1 M as a supporting electrolyte. The free energy change ∆G_et_ for an electron transfer reaction was calculated from the classical Rehm–Weller equation (Equation (1)), where E_ox_, E_red_, E*, and C are the oxidation potential of the electron donor, the reduction potential of the electron acceptor, the excited state energy level, and the coulombic term for the initially formed ion pair, respectively [37]. C is neglected, as is usually in polar solvents.

∆G_et_ = E_ox_ − E_red_ − E* + C. (1)

### 2.5. Scanning Image Macroscope (SEM)

The morphology and particle size of the polymer was examined by a field emission scanning electron microscope (FESEM) (JEOL JSM 6490-A) at different resolutions.

### 2.6. Fluorescence Experiments

The fluorescence properties of the copper complex (HLCuCl) were determined in DMF using a JASCO FP-8200 spectrometer (JASCO, Riyadh, Saudi Arabia).

## 3. Results

### 3.1. Characterization of the Ligand and Its Copper Complex

The ligand and the synthesized copper chloride complex were stable at room temperature, freely soluble in DMF, non-hygroscopic in nature, and analyzed on the basis of various spectroscopic techniques to determine their structure. The results of the elemental analysis were in agreement with those of the proposed structure of the synthesized compounds. The observed low conductance values suggested their non-electrolytic nature.

#### 3.1.1. Mass Spectrum

**Scheme 3:**
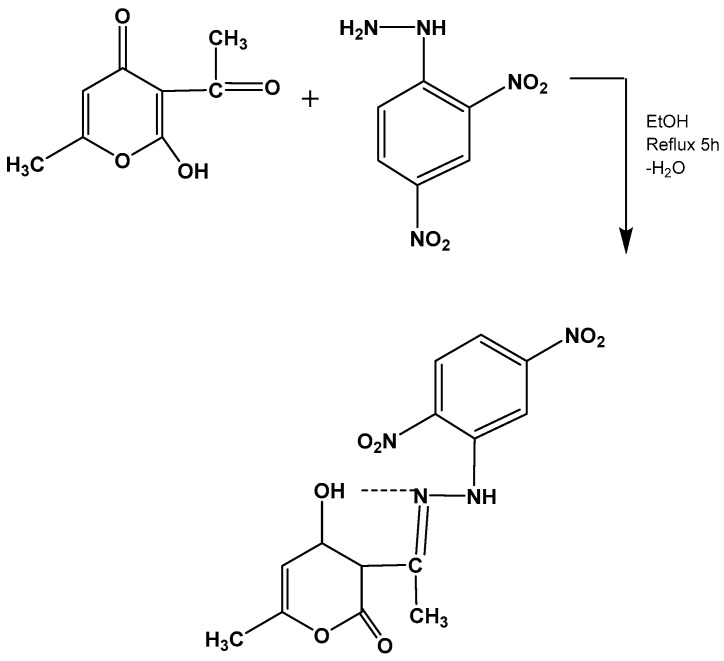
Synthesis of the ligand (HL).

**Scheme 4:**
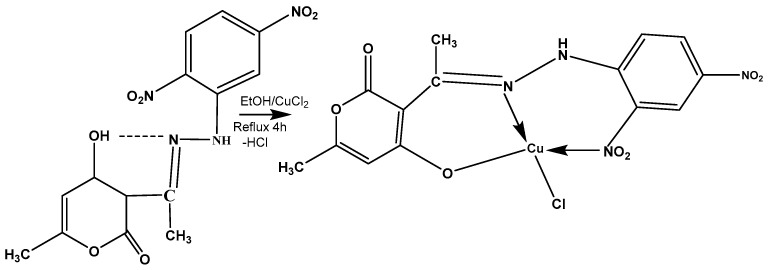
Synthesis of the complex (HLCuCl).

The mass spectrum of the ligand showed an intense molecular ion peak at m/z 348, indicating the formation of a desired compound, as suggested in (Scheme 3 and Scheme 4). Other prominent peaks were observed at m/z 313,306,291,267,260, 245,227, 219,198,181,167,151,126,115,109,85,77,67, and 35, respectively (See Figure S1).

#### 3.1.2. 1H NMR Spectrum

The 1H NMR spectrum of the ligand displayed signals at 2.26 (singlet, 3H) due to –CH_3_ proton, 2.45 (singlet, 3H) due to –N=C–CH_3_, 6.21 (singlet, 1H) due to the olefinic proton of heterocyclic DHA ring. and at 9.0 and 10.9 ppm (singlet, 1H) due to the NH and enolic OH protons, respectively. The very downfield value of enol 1 H NMR indicates a very strong intramolecular hydrogen bonding O–H…N. The resonance stabilization of these compounds favours the enol form [38]. The multiple signals appeared at 8.4–7.8 ppm due to an aromatic ring (m, Ar-CH phenyl [39]; Figure S2).

#### 3.1.3. FTIR IR Spectra

The FTIR spectrum of the copper complex was compared with that of the free ligand in order to investigate the mode and nature of the chelation of a metal ion with a ligand. In the FTIR spectrum, the free ligand showed some characteristic bands at 3441, 3098, 2926, and 2852 m, and at 2922, 1716, and 1613 cm^−1^ (see Figure S3) due to OH (intramolecular hydrogen bonding), N–H (stretch), 2C–H (stretch), C=O (carbonyl), and C=N (azomethine) stretching, respectively [40]. In the copper complex, the disappearance of a band centered at 3441cm^−1^ suggests the deprotonation of enolic proton and the involvement of the enolic oxygen atom in the coordination with the copper ion. Increasing the frequency of the C=N band at 1614 cm^−1^ compared to that of the free ligand indicates the involvement of the azomethine group in coordination with the copper ion [41], suggesting that copper ions coordinate through the oxygen atom and the nitrogen of the atom azomethine group [40]. Moreover, the spectrum consists of three non-ligand bands at 555, 517, and 449cm^−1^ which can be assigned to the Cu–O, Cu–N, and Cu–Cl vibrations, respectively [42]. The ligand HL behaved as monobasic tridentate towards cupric ions. The proposed structures of the copper complex are presented in Figure S4.

#### 3.1.4. The DTA and TGA Spectra

The diagram of the ligand is illustrated in Figure S2. The DTA diagram shows an endothermic peak at 132.28 °C with no weight lost, as also indicated by the TGA curve due to the melting of the compound. The exothermic peak appeared at 238.91 °C in the DTA curve and started at 219.92 °C and ended at 300.7 °C in the TGA curve, with a weight loss of 37.77%, and may be assigned to losses of para nitro aniline. Another two peaks appeared at 317.15 and 444.43 °C in the DTA curve and in the TGA curve at 300.73–411 and 411–1001.35 °C, with a weight loss of 16.02% and 25.82%, respectively, and were assigned to the chemical decomposition of organic compound; see Figure S5.

### 3.2. Light Absorption and Magnetic Properties of Cu-Complex

**Figure 1:**
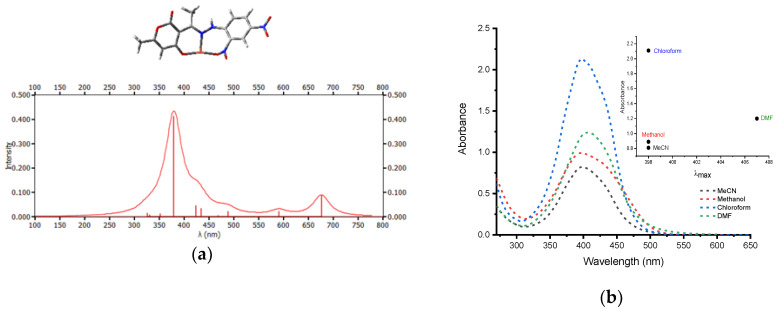
Optimized (by the UB3LYP/6-31G* method) (**a**) copper complex geometries and their UV–Vis spectra; (**b**) UV–Visible spectra of HLCuCl complex in MeCN, methanol, and chloroform, and in a DMF concentration of 10^−4^ M.

The calculated UV–Vis absorption (at the UB3LYP/6-31G* method) of the copper complex is plotted. In Figure 1a, this last UV-vis transition is compared with the actual experimental UV–Vis spectra in Figure 1b.

The UV–Vis. electronic spectra of the copper II complex in four organic solvents with different polarities were also studied. The UV–Vis. absorption spectra of the studied complex were measured in dimethylformamide, ethanol, chloroform, and acetonitrile. Figure 1b shows the absorption spectra of a 10^−4^ mol L^−1^ solution of HLCuCl in these solvents as a sample. As can be seen from Figure 1b, all the solvents tested the main band of the studied compound, which was located in the spectral range of 398–407 nm. This band is characteristic of ligand–metal charge transfer transition (LMCT) [43,44,45]. In the UV–Visible region observed from the studied compound in different solvent systems, this is assigned to the π→π* transition of the C=N [46]. The copper II complex is characterized by high molar extinction coefficients in the different solvents (see Table 1).

The observed band and the magnetic moment 1.72 B.M. of copper (II) complex are close to the spin-only value of one unpaired electron, indicating a square planar geometry around the Cu(II) ion [47] and suggesting a square-planar geometry around the copper ion [48,49].

**Figure 2:**
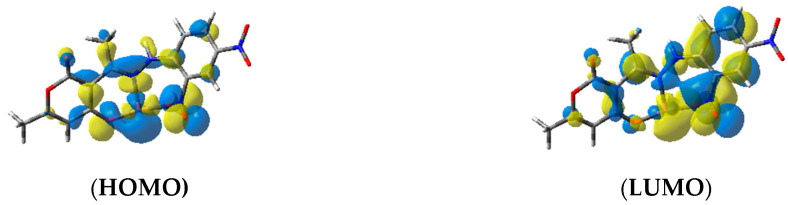
Contour plots of HOMOs and LUMOs for the copper complex structure optimized at the B3LYP/6-31G* level of theory.

The HOMO and LUMO calculated from molecular orbital calculations well show this п-п* transition character (Figure 2) with frontier orbitals well-delocalized on the ligand with a participation of the Cu center.

### 3.3. Copper Complex Oxidation Process

The oxidation potential of E_ox_ from the copper complex is presented in Table 2 in acetonitrile. It was evaluated by measuring the cyclic voltammetry with tetrabutyl-ammonium hexafluorophosphate 0.1 M as a supporting electrolyte. issolved oxygen was removed by bubbling nitrogen gas (see Figure S6.). The excited state energies were evaluated from the crossing point of the absorption and luminescence spectra (see Figure S7).

The free energy change ∆G_et_ for the electron transfer reaction between the copper complex and *diphenyliodonium hexafluorophosphate* (Iod), TEA, or gold chloride was calculated from the classical Rehm–Weller equation (Equation (1)) [34]. The free energy change of the HLCuCl/Iod electron transfer reaction ∆G_et_ = −1.16 eV is highly negative and make the process favorable (using E_red_ (Iod) = −0.2 eV) [50]. The counteranions of iodonium salts would not affect the results of the photochemical mechanism studies for the initiation process. For the reaction between the copper complex and the triethylamine, it is ∆G_et_ = −0.82 eV, which also supports a favorable electron transfer process (using E_ox_ = 1.079 V for the triethylamine) [51].

### 3.4. Photoinduced Synthsis of Au Nanoparticles

Noble metals are known to exhibit unique optical properties due to their property of Surface Plasmon Resonance (SPR), which is the collective oscillation of the conduction electrons in resonance with the wavelength of the irradiated light. In the present study, the formation of gold nanoparticles was initially conformed using UV–Visible spectroscopy by measuring the SPR peaks. Gold nanoparticles exhibit plasmon absorption bands that depend on their size and shape.

We report the use of triethylamine as reducing agents in the formation of gold nanoparticles in the presence of a copper complex and iodonium salt. The study of Newman et al. [51] indicates the utility of amines as reducing agents in the formation of AuNPs and provides information on the conditions under which these reactions will take place.

**Figure 3:**
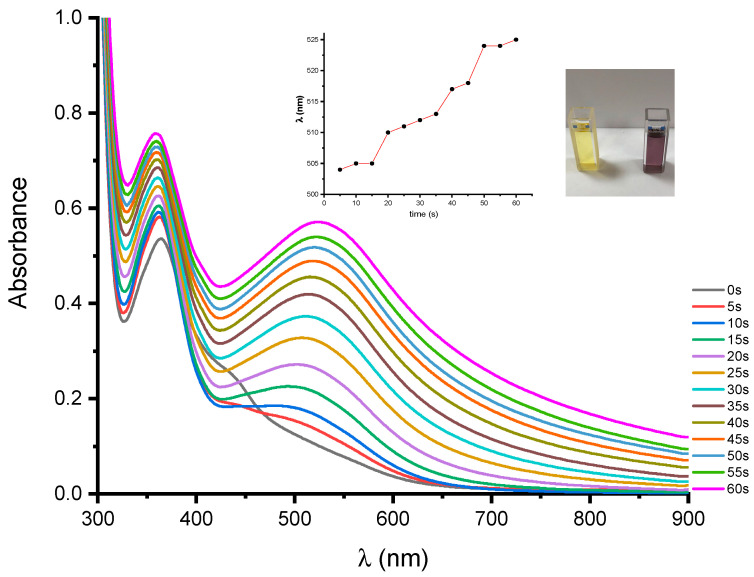
Evolution of the absorption spectra of the irradiated mixtures (λirr = 419 nm). Solution: HLCuCl 0.05 wt % gold chloride 4wt% and TEA 1 wt% dissolved in 25 mL of DMF.

For the synthesis of gold nanoparticles, 1 mL of TEA was added to 1 mL of gold chloride and 1 mL of HLCuCl solution in DMF. The sample was stirred continuously for 5 min. at room temperature. The HLCuCl/TEA/HAuCl_4_ system shows the increase in the absorption at 362 nm during irradiation, with the appearance of a peak at 525 nm. The color gradually changed from yellow to dark purple within a minute of irradiation (see Figure 3). The absorption at 525 nm corresponding with the SPR peak and the change in colors and characteristics of the surface plasmon absorption spectra indicates the formation of Au nanoparticles with the size of 30 nm [52,53].

**Scheme 5:**
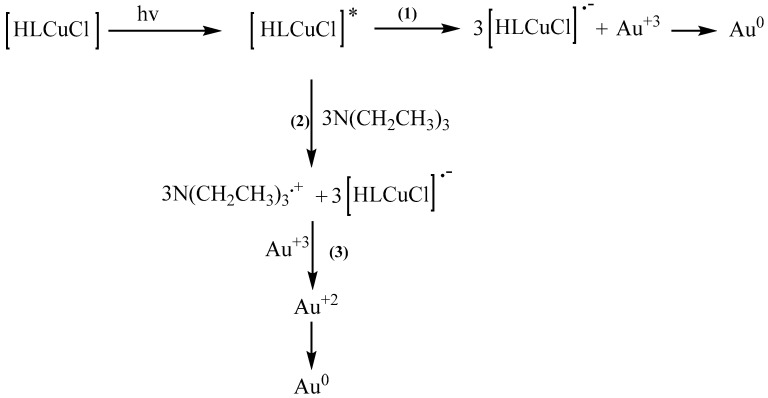
Proposed mechanism of the system based on HLCuCl/TEA.

The inserts in Figure 3 show the time traces for the maximum position of the plasmon band for the system based on copper complexes and triethylamine; a clear red shift is observed during the initial stages of the process. The interaction of the copper complex, triethylamine, and gold chloride was very fast, being within 60 s. A small amount of copper complex was sufficient to generate radical ions, which reduce the tetrachloroaurate to form Au^+2^. This is disproportionate to Au^+3^, which is then reduced to Au^0^, leading to the formation of nanoparticles (see Figure S8). Moreover, the reduction of HAuCl_4_ occurs due to a transfer of electrons from the amine to the metal ion, resulting in the formation of Au^0^, with the reaction being generically described according to Scheme 5.

**Scheme 6:**
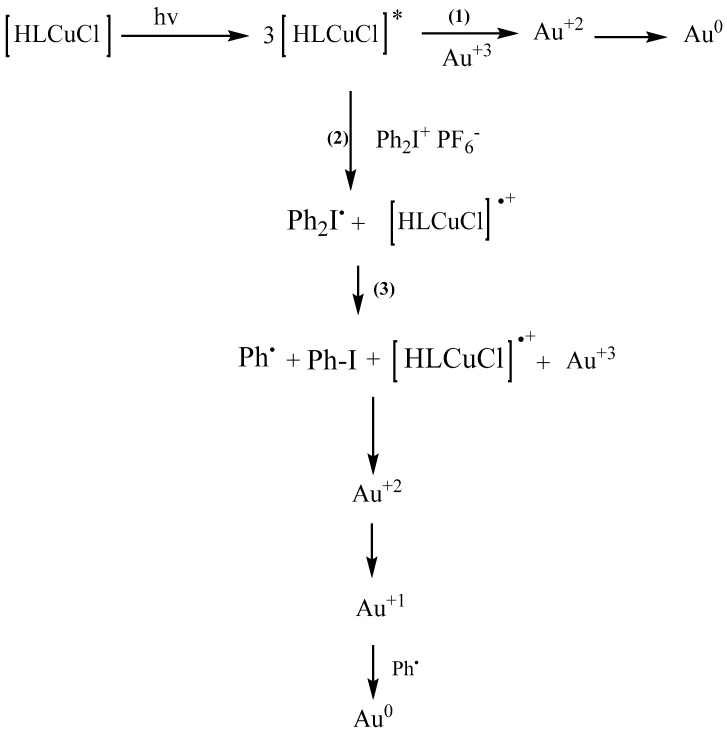
Proposal mechanism of the system based on HLCuCl/Iod salt.

**Figure 4:**
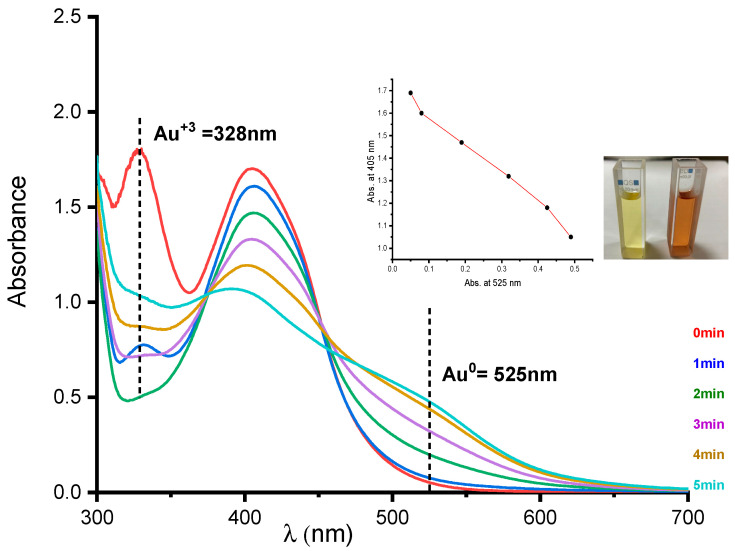
Evolution of the absorption spectra of irradiated mixtures (λirr = 419 nm). Solution: HLCuCl 0.05 wt% gold chloride 4wt% and iodonium salt 1 wt% dissolved in 25 mL of DMF.

The system which contains copper II complex 0.05 wt% gold (III), chloride 4 wt%, and Iodonium salt 1 wt% dissolved in 25 mL of DMF shows better efficiency in producing gold nanoparticles under visible irradiation at 419 nm, with an intensity of 250 microwatts/cm^2^. Figure 4 shows the appearance of the absorption band of Au^+3^ at 328 nm [54] at t = 0 s, and also the maximum absorption of the HLCuCl complex at 407 nm after one minute of irradiation, where we have seen a large decrease in the band at 328 nm and the change in the color to orange. After five minutes under irradiation, the absorption of the complex and Au^+3^ have entirely disappeared; we notice the increase in absorption at 525 nm, which corresponds the surface plasmon resonance (SPR) absorption. A careful analysis of the absorption spectrum during the irradiation period clearly reveals the following:

- The first step corresponds to the photobleaching of HLCuCl with the concomitant growth of the surface plasmon band, as indicated by the linear correlation between the absorbance at 407 nm vs. the absorbance at 525 nm (inset in Figure 4).
- In a second step, the decomposition of the iodonium salt through an electron transfer and the production of a phenyl radical that is able to abstract hydrogen to generate radicals reduces the Au^+3^ to Au^+2^. The Au^+2^ is unstable and can be reduced with the radical to Au^+1^ [12]. Then, the Au^+1^ can be reduced by another radical to Au (0). Scheme 6 illustrates the proposed reaction mechanism.

**Figure 5:**
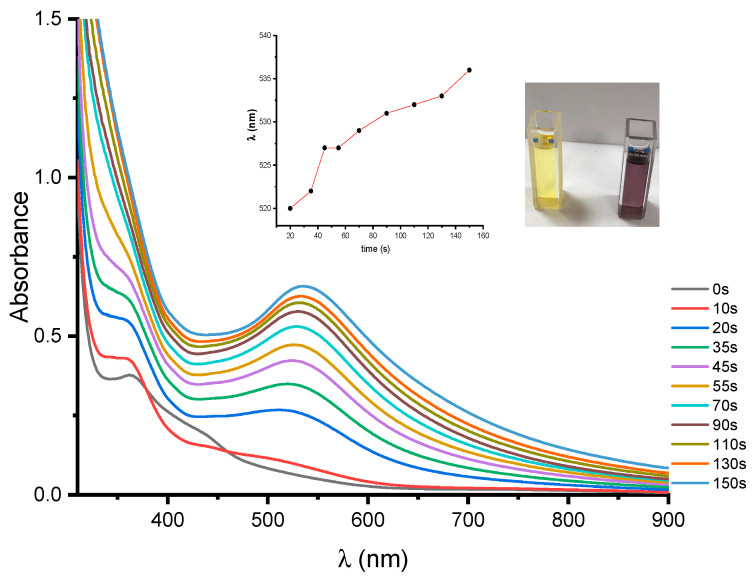
Evolution of the absorption spectra of irradiated mixtures (λirr = 419 nm). Solution: HLCuCl 0.05 wt% gold chloride 4wt%, TEA 1wt%, and iodonium salt 1 wt% dissolved in 25 mL of DMF.

Figure 5 illustrates the UV–Vis absorption spectra of the PIS system HLCuCl/TEA/Iod solution obtained during irradiation in the presence of gold chloride. The formation of gold nanoparticles in the PIS take places through the reduction of Au^3+^ to Au^0^, and this outcome can be proven by the appearance of characteristic plasmons in the UV–Vis absorption spectra at 536 nm. The peak has a symmetrical shape, indicating the uniform size distribution of the gold nanoparticles in the organic phase [55]. The insert in Figure 5 shows that the SPR band changes with the irradiation time, and it was also noticed that the violet color grew increasingly darker with the radiation time. These results show that a simple irradiation of a copper complex in the presence of HAuCl4 leads to its reduction, with the rapid generation of both metallic gold and initiating radicals without any undesirable side reactions.

### 3.5. Fabrication of AuNPS Embedded Polymer

Metal ions are reduced inside a polymer matrix. This technique leads to a homogeneous dispersion of the metallic nanoparticles in the polymer matrix. The in situ process to prepare and stabilize nanoparticles can be combined in one step. This process gives the opportunity of a quick and easy way to synthesize stabilized NPs.

The UV-crosslinked irradiated for 10 min under air were subjected to UV–Vis spectroscopy analyses. The sample consists of HLCuCl/Iod/TEA as the photoinitiating system in the presence of gold chloride, and EGDA was used as a monomer. The photopolymerization formulation was prepared by HLCuCl 0.05 wt% gold chloride 4 wt% in a few drops of DMF, TEA 1 wt%, and iodonium salt 1 wt% dissolved in EGDA at 93.95 wt%. The last was stirred continuously overnight at room temperature.

**Figure 6:**
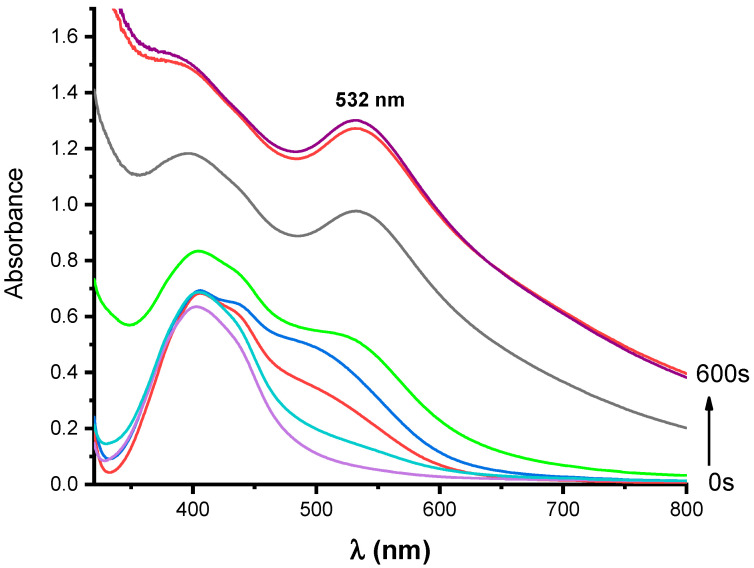
Time evolution of the absorption spectrum of the ethylene glycol diacrylate (EGDA) during photopolymerization, irradiated mixtures (λ_irr_ = 419 nm). Solution: HLCuCl 0.05 wt% gold chloride 4wt%, TEA 1wt%, and iodonium salt 1 wt% dissolved in 93.95 wt%.

The spectra in Figure 6 show a clear and strong absorption band centered at 532 nm and another peak at 407 nm, as had happened for the photoinitiating system solutions HLCuCl/Iod/TEA in the presence of HAuCl_4_ in the spectra of Figure 5. The intensity of the band is similar to that seen in the spectra of Figure 6. The strong absorption peak at 532 nm can be attributed to the formation of plasmon resonance gold nanoparticles, as was previously recorded for the UV irradiation of a photoinitiating system containing the gold ion. The peak at 407 nm is related to the HLCuCl. The formation of the narrow SPR band of AuNPs clearly confirms the formation of Au nanoparticles in the cross-linked acrylate formulation by UV irradiation. The color changes of these gels from yellow to dark purple also represent unambiguous proof in favor of the AuNPs development and deposition within the polymeric template. The color changes in these gels range from a transparent color to a purple-colored form depending on the irradiation time.

**Figure 7:**
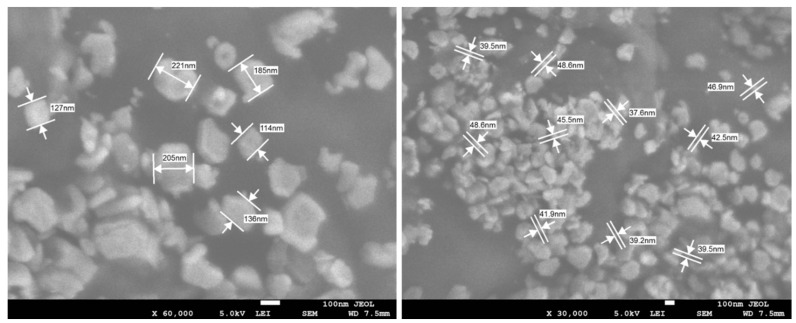
SEM image of gold nanoparticles (AuNPs) in the polymer matrix HLCuCl 0.05 wt% gold chloride 4 wt%, TEA 1wt%, and iodonium salt 1 wt% dissolved in EGDA 93.95 wt%.

SEM measurement also supported the controlled size distribution of AuNPs without agglomeration; see Figure 7. The increase in the density of the cross-links of the nanocomposite material led to the formation of large-sized AuNPs. The SEM images and SPR absorbance of fhe AuNPs support this (see Figure 6 and Figure 7). The shape of AuNPs is mainly spherical, and the size is controlled. The size, shape, and distribution of AuNPs were facilitated by HLCuCl, but the ethylene glycol-based acrylic monomer also contributed to this due to the chelation effect [22]. A difference in size is observed with ranges between 37 and 200 nm (see Figure 7). The overall crosslink efficacy depends on the UV light intensity, the dose (or irradiation time), and the concentration of the initiator and gold chloride. The selected relative concentration of 0.05 wt%, 4 wt%, and 1 wt% have not been optimized. Furthermore, a higher UV light intensity will accelerate the crosslink; however, it might also reduce the efficacy. Greater background may be found in the kinetic models of Lin et al. [56,57].

**Figure 8:**
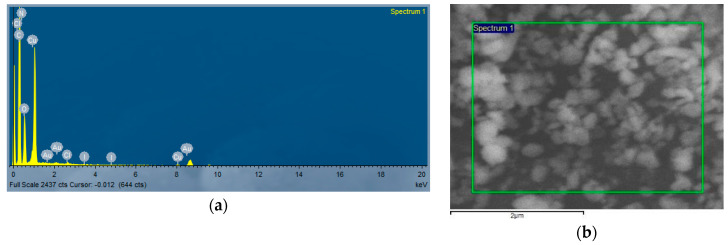
EDX graph of the polymer matrix (**a**), SEM image of the AuNPs in the polymer matrix (**b**).

All the hybrid nanocomposites with Au NPs formed in situ were explored by means of energy-dispersive spectroscopy (EDS), a technique that permits the quantification of the amount of material. In the EDS patterns (Figure 8), the characteristic peaks for C, O, N, Cl, I, and Cu along with the signal specific for elemental gold indicate that the reduction of gold ions to Au^0^ took place, thus supporting the results presented earlier. Hybrid nanocomposites with Au NPs formed in situ were explored by means of energy-dispersive spectroscopy (EDS), a technique that permits the quantification of the amount of material. In the EDS patterns (Figure 8), the characteristic peaks for C, O, N, Cl, I, and Cu along with the signal specific for elemental gold indicate that the reduction of gold ions to Au^0^ took place, thus supporting the results presented earlier.

## 4. Conclusions

In this paper, a new copper complex based on a hydrazone ligand was proposed as a photocatalyst for the polymerization of ethylene glycol diacrylate simultaneously with the production of gold nanoparticles in a polymer network under exposure to an LED at 419 nm. The short irradiation of the mixture containing copper complexes with polymerizable acrylate groups (EGDA) and HAuCl_4_ in the presence of triethylamine and iodonium salt results in the formation of the desired composites with a good size of gold NPs (37–200 nm). The method described here is quite interesting because it allows the simultaneous formation of gold nanoparticles and a lattice which secures the resulting structure and allows long-term stability. The morphology of the hardened systems, studied by SEM analysis, has shown that the nanoparticles are distributed homogeneously.

## Figures and Tables

**Table 1:** Molar extinction coefficients ε in different solvents for the cu-complex investigated.

ε (M^−1^ cm^−1^)	λ_max_ (nm)	Solvents
8270	398	Acetonitrile
9900	398	Methanol
12,390	407	DMF
21,260	398	Chloroform

**Table 2:** Excited state energies E*, oxidation potentials E_ox_, and free energy change (∆G_et_) for the HLCuCl/TEA/Iod interaction.

E_ox_ vs. SCE* [V] [HLCuCl]	E^*^[eV] [HLCuCl]	∆G_et_ (HLCuCl II /Iod) (eV)	∆G_et_ (HLCuCl II /TEA) (eV)
1.44	2.8	−1.16	−0.82

## Supplementary Materials

The following are available online at https://www.mdpi.com/2073-4360/12/10/2293/s1: Figure S1: Mass spectrum of the ligand (HL). Figure S2: H1NMR spectrum of the ligand (HL). Figure S3: IR spectrum of the ligand (HL). Figure S4: IR spectrum of the copper complex. Figure S5. DTA, TGA and DrTGA of the ligand (HL). Figure S6: Cyclic voltammogram of HLCuCl in acetonitrile. Figure S7: Photoluminescence of HLCuCl in DMF. Figure S8. Evolution of the absorption spectra of irradiated mixtures (λirr = 419 nm). Solution: HLCuCl 0.05 wt% and gold chloride 4wt% dissolved in 25 mL of DMF.
