# Supplementary material for: Novel Copper Photoredox Catalysts for Polymerization: An In Situ Synthesis of Metal Nanoparticles
Source: Polymers (Basel). 2020 Oct 7;12(10):2293. doi: 10.3390/polym12102293 (PMC7599841; doi:10.3390/polym12102293)
Supplement: Supplementary file 1 [file polymers-12-02293-s001.pdf]

Supporting Information

Novel Copper Photoredox Catalysts for Polymerization: In-situ Synthesis of Metal Nanoparticles

Haja Tar [1,*], Tahani I. Kashar [2], Noura Kouki[1], Reema Aldawas [1], Bernadette Graff[3] and Jacques Lalevée[3]

[1] Department of Chemistry, College of Science, Qassim University, King Abdulaziz Rd, Buraydah, Qassim 1162 SA, Saudi Arabia; n.kouki@qu.edu.sa (N.K.), Rdoas@qu.edu.sa (R.A.)
[2] Department of Chemistry, Faculty of Science, Menoufia University, Shebin El-Kom, Egypt; tahanikashar@yahoo.com (T.I.K.)
[3] Institut de Science des Matériaux de Mulhouse IS2M – UMR CNRS 7361 – UHA, 15, rue Jean Starcky, 68057 Mulhouse Cedex, France ; jacques.lalevee@uha.fr (J.L.) ; bernadette.graff@uha.fr (B.G.)
* Correspondence: h.tar@qu.edu.sa (H.T); Tel.: +(966) 16-38-13490 (H.T.)

Experimental part

All reagents and solvents were purchased from Aldrich and used as received without further purification. Elemental analyses (EA) (C, H, N and Cl) were determined using atomic absorption with a Perkin-Elmer 2380 spectrophotometer. The IR spectra using a Perkin-Elmer 1430 infrared spectrometer were measured as KBr discs in range 4000-200 cm^{-1}. Electronic absorption spectra in the 200-900 nm region were recorded on a Perkin-Elmer 550 spectro-photometer. The Gouy method was used to measure magnetic susceptibilities at room temperature. A Bibby conductometer MCl was used for conductance measurements. Thermal analyses (TGA/DTG) were carried out by using a Shimadzu DTG/TG-50 thermal Analyzer with a heating rate of 10°C/min in nitrogen atmosphere with a flowing rate of 20/ml.

Synthesis of 3-Hydroxy-N′-(1-(6-methyl-2,4-dioxo-3,4-dihydro-2H-pyran-3-yl)ethylidene)-2,4 dinitrophenylhydrazone (HL)

A 30ml ethanolic solution 3-acetyl-2-hydroxy-6-methyl-4H-pyran-4-one (DHA) (0.01mol) was added to an equimolar ethanolic solution of 2,4 dinitrophenylhydrazine (0.01mol) dropwise and refluxed for 5 hr. The resulting pale red colored precipitates were filtered and dried in a vacuum desiccator over anhydrous calcium chloride Scheme (1).Yield: 90%; m.p: °C; Selected IR data (KBr,v/cm−1): 3441 cm^{-1}b (O-H str), 3098 m(N-H str), 2926 cm^{-1} m (aromatic C-H str), 2852 m, 2922 m v (aliphatic C-H str), 1716 cm^{-1}s v(C=O str), 1613 cm^{-1}s v(C=N), 1H NMR (400MHz, DMSO-d6): 2.26 (s, CH_3), 2.45 (s, CH_3), 6.21 (s, CH arm.), 8.4–7.8 (m, Ar-CH phenyl, 9.0(s,NH),10.9 (s, OH) ppm. ESI MS(m/z):348,333,313,306,291,267,260,245,227,219,198, 181,167,151,126,115,109,85,77,67 and 35. Anal. Calc. for $C_{14}H_{12}O_7N_4$ (348): C, 48.27; H, 3.45; N, 16.0. Found: C, 48.27; H, 3.31; N, 15.94%.

Synthesis of copper II complex of 3-hydroxy- N′-(1-(6-methyl-2,4-dioxo-3,4-dihydro-2H-pyran-3-yl) ethylidene) 2,4 dinitrophenylhydrazone [HLCuCl]

The copper complex was prepared by reacting 1:1 stoichiometric ratio of the ligand and copper chloride. An ethanolic solution of the respective copper chloride (0.01 mol) was added to 15 ml ethanolic solution of the hydrazine (H_2L) (0.01mol) while being stirred. The reaction was refluxed for 4hr. The resulting precipitates were filtered off, washed with cold ethanol and dried in vacuum desiccator over anhydrous calcium chloride. Yield: 80%; m.p: 130°C; Selected IR data (KBr,v/cm−1): 3445 cm^{-1}b (O-H str),3165 cm^{-1} m(N-H str), 2927 cm^{-1} m (aromatic C-H str), 2927 cm^{-1} m (aromatic C-H str), 1713 cm^{-1}s v(C=O str), 1614 cm^{-1}s v(C=N), 555 cm^{-1}m(Cu-O), 517 cm^{-1}m(Cu-N), 449 cm^{-1}w(Cu-Cl). Anal. Calc. for Cu $C_{14}H_{11}O_7N_4$ Cl (447): C, 37.6; H, 2,5; N,12. 5. Found: C,37.1;H,3.1;N,12.2 μeff (BM): 1.73. Molar conductance (Ω−1cm2mol−1): 10.3.

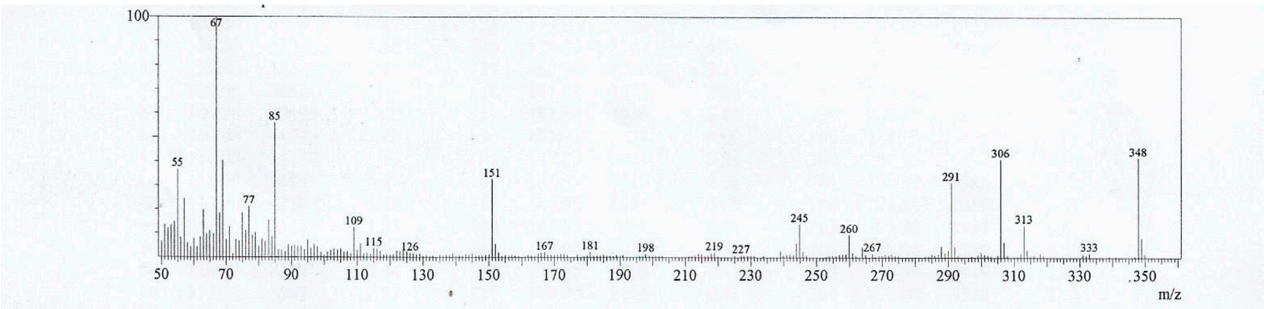

Figure S1. Mass spectrum of the ligand (HL)

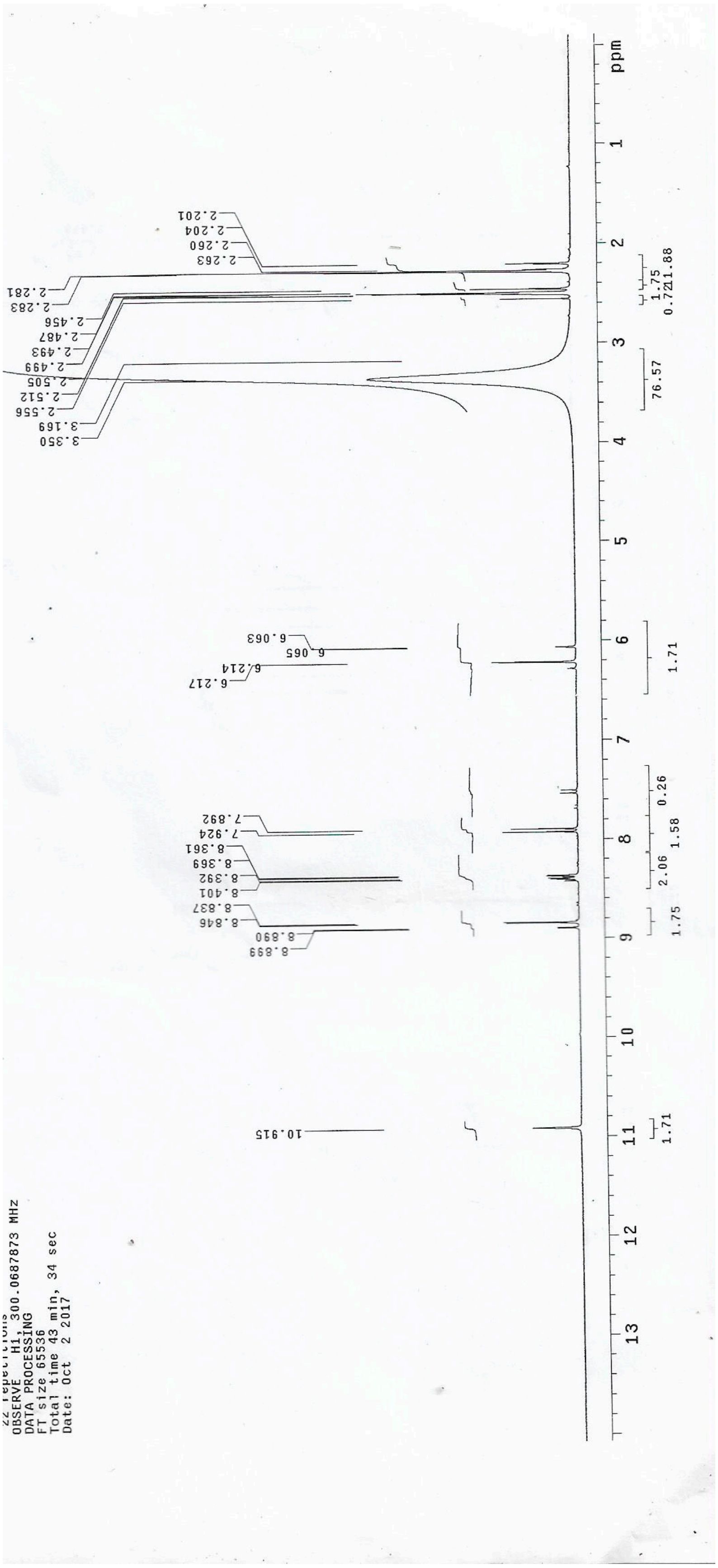

Figure S2. H^1NMR spectrum of the ligand (HL)

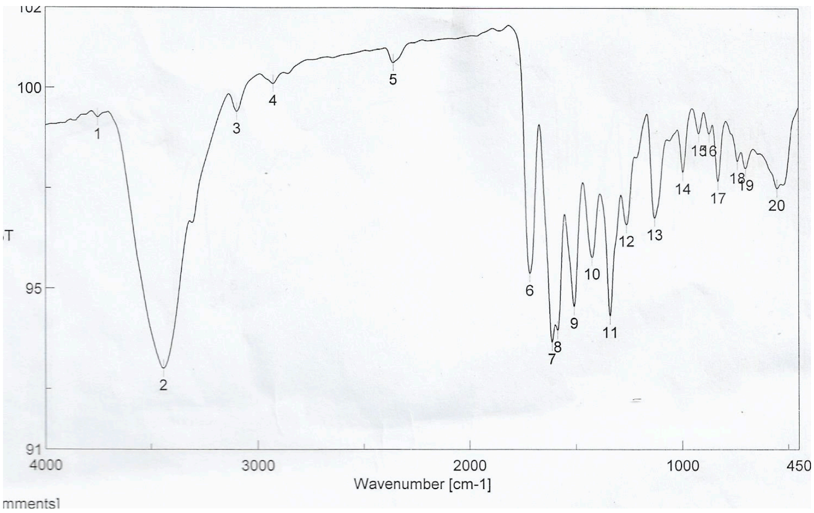

Figure S3. IR .spectrum of the ligand (HL)

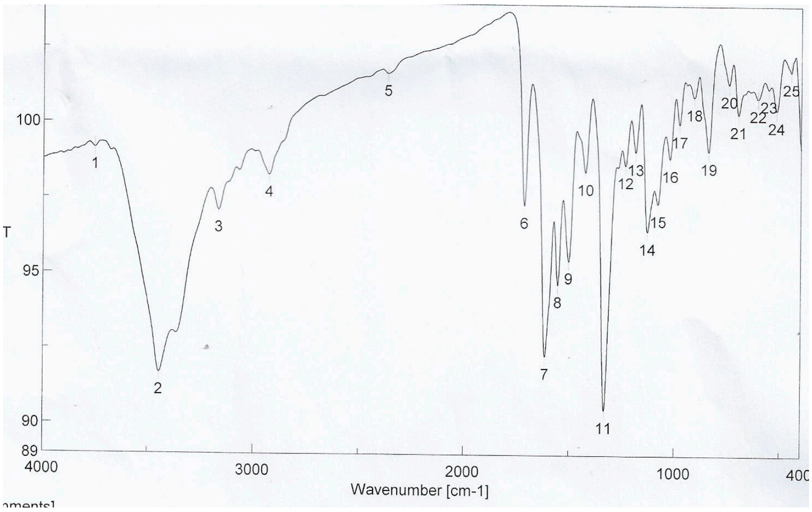

Figure S4. IR .spectrum of the copper complex

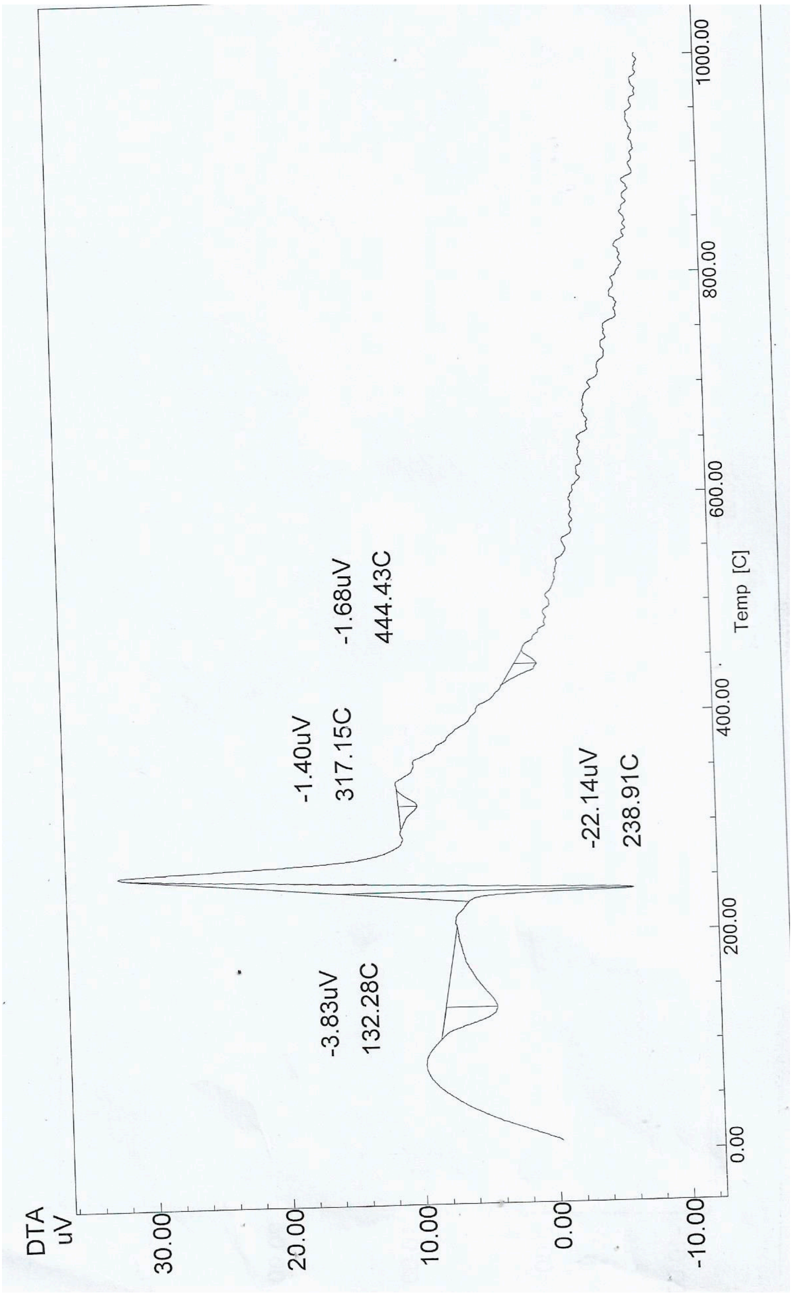
DTA
uV
30.00
20.00
10.00
0.00
-10.00
0.00
200.00
400.00
600.00
800.00
1000.00
Temp [C]
-3.83uV
132.28C
-22.14uV
238.91C
-1.40uV
317.15C
-1.68uV
444.43C

(**a**)

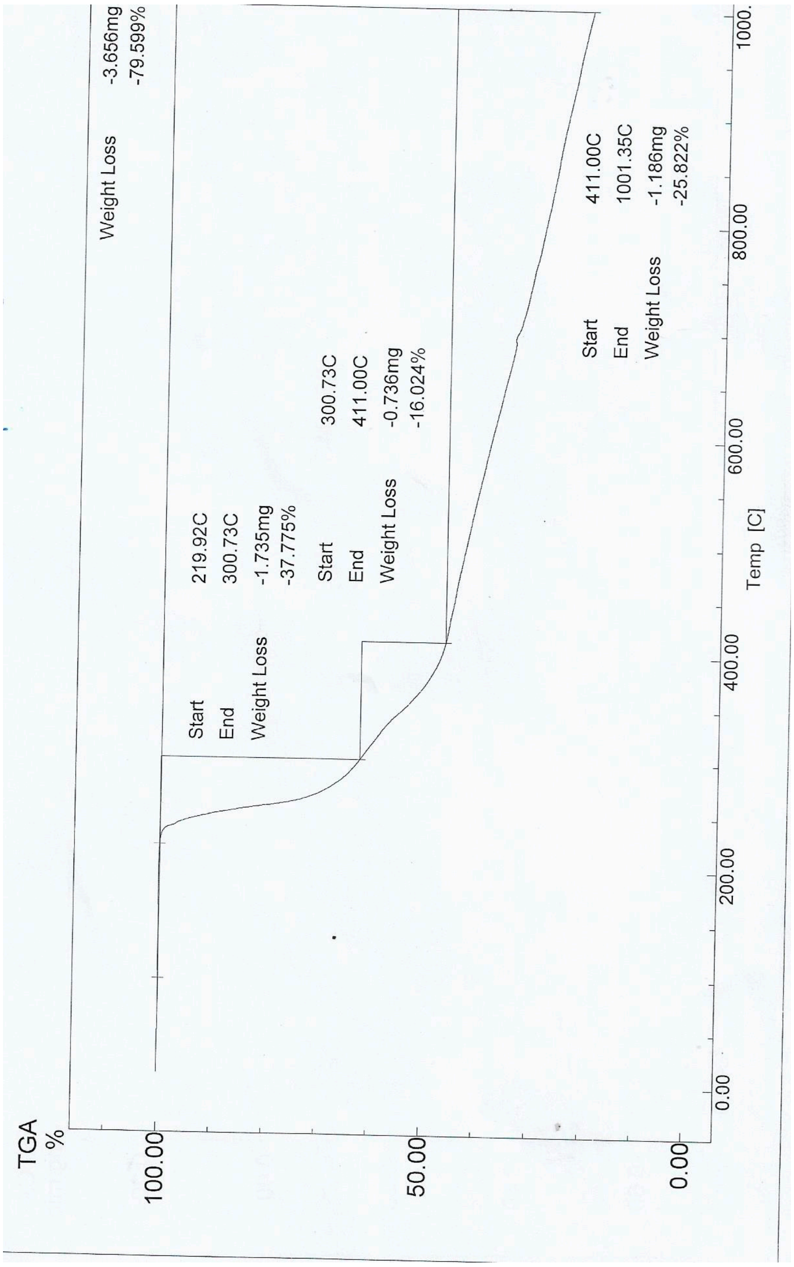

TGA
%
100.00
50.00
0.00
0.00
200.00
400.00
600.00
800.00
1000.
Temp [C]
Weight Loss
-3.656mg
-79.599%
Start
End
Weight Loss
219.92C
300.73C
-1.735mg
-37.775%
Start
End
Weight Loss
300.73C
411.00C
-0.736mg
-16.024%
Start
End
Weight Loss
411.00C
1001.35C
-1.186mg
-25.822%

(**b**)

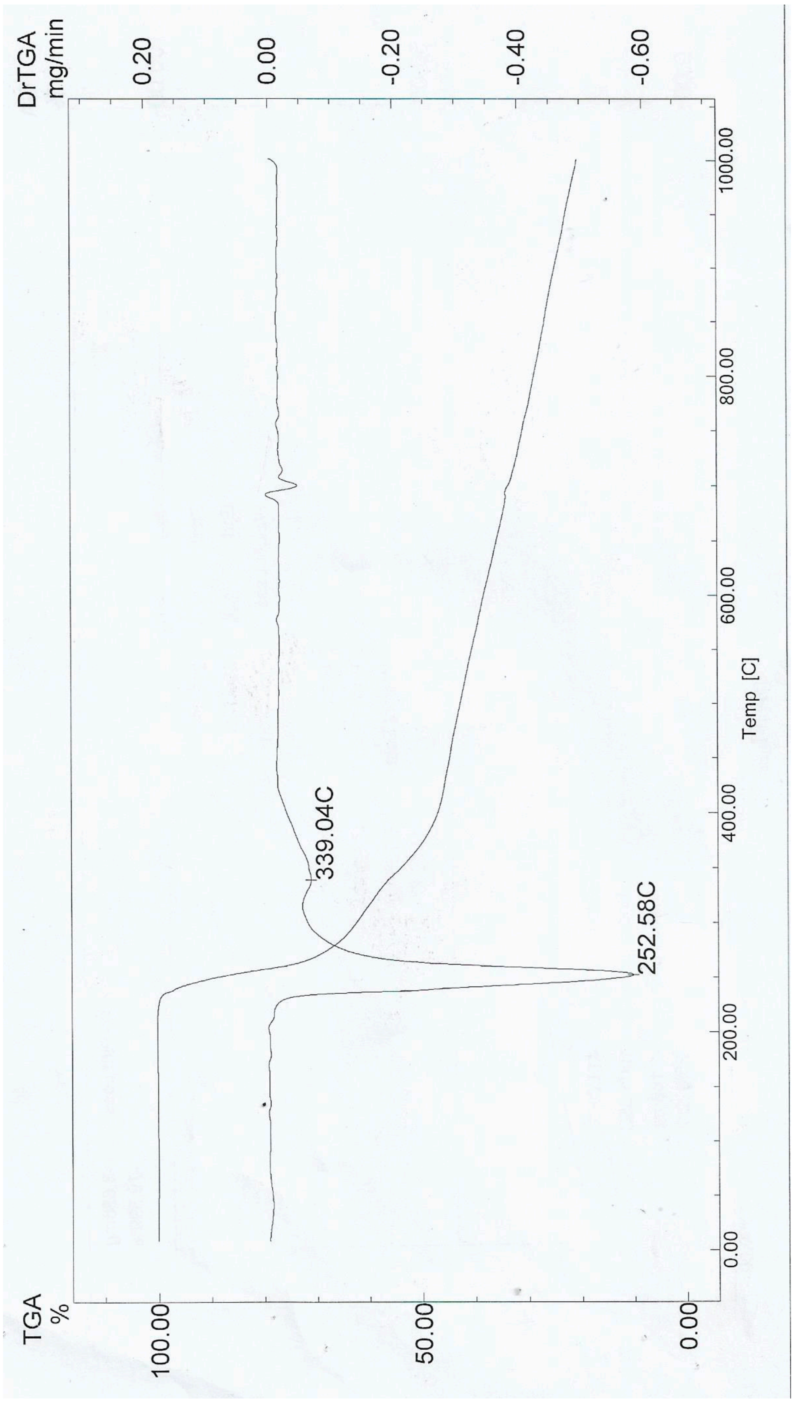

(**c**)

Figure S5. DTA, TGA and DrTGA of the ligand (HL).

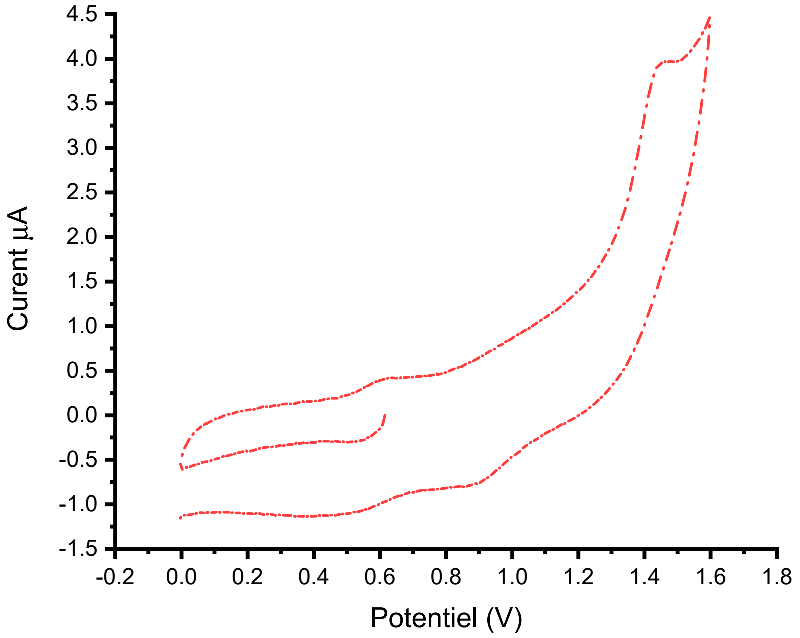

Figure S6. Cyclic voltammogram of HLCuCl in acetonitrile.

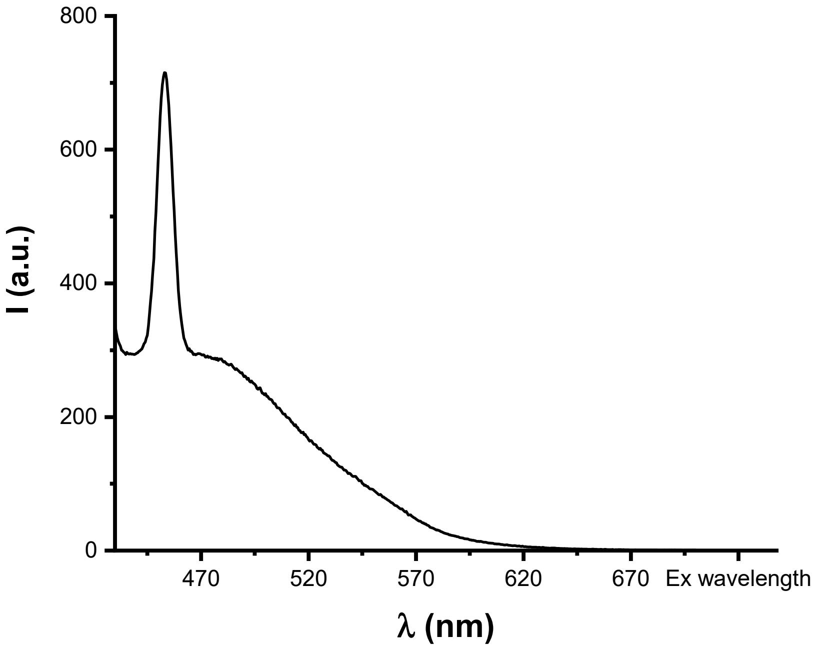

Figure S7. Photoluminescence of HLCuCl in DMF

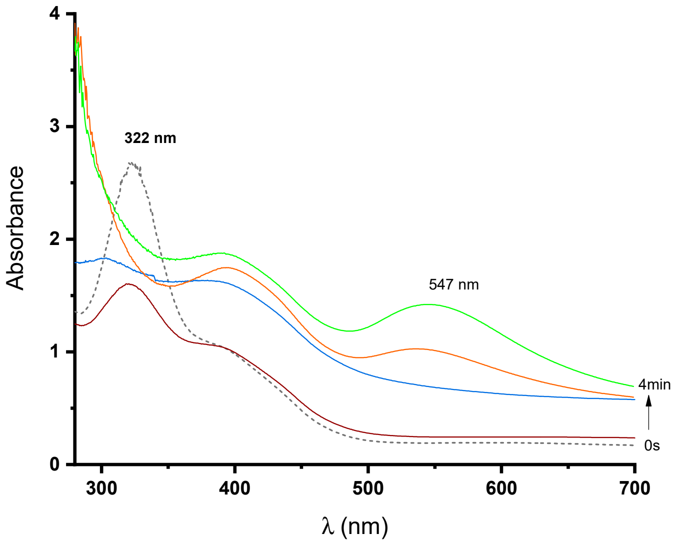

Figure S8. Evolution of the absorption spectra of irradiated mixtures (λ_{irr} = 419 nm). Solution: HLCuCl 0.05 wt% and gold Chloride 4wt% dissolved in 25 ml DMF.
